# KRAS G12C Inhibition in Solid Tumors: Biological Breakthroughs, Clinical Evidence, and Open Challenges

**DOI:** 10.3390/cancers17172803

**Published:** 2025-08-27

**Authors:** Pietro Paolo Vitiello, Anna Amela Valsecchi, Eleonora Duregon, Paola Francia Di Celle, Paola Cassoni, Mauro Papotti, Alberto Bardelli, Massimo Di Maio

**Affiliations:** 1 Department of Oncology, A.O.U. Città della Salute e della Scienza di Torino, University of Turin, Ospedale Molinette, 10126 Turin, Italy; dr.ssavalsecchianna@gmail.com (A.A.V.); massimo.dimaio@unito.it (M.D.M.); 2 IFOM ETS, The AIRC Institute of Molecular Oncology, 20139 Milan, Italy; 3 Molecular Biotechnology Center (MBC), Department of Oncology, University of Turin, 10126 Turin, Italy; 4 Pathology Unit, Department of Oncology, A.O.U. Città della Salute e della Scienza di Torino, University of Turin, 10126 Turin, Italy; 5 Molecular Pathology, A.O.U. Città della Salute e della Scienza di Torino, 10126 Turin, Italy; 6 Pathology Unit, Department of Medical Sciences, A.O.U. Città della Salute e della Scienza di Torino, University of Turin, 10126 Turin, Italy

**Keywords:** KRAS G12C, targeted therapy, translational oncology

## Abstract

KRAS is the most frequently mutated oncogene in cancer. Its activating mutations are associated with aggressive tumor behavior and resistance to certain therapies, including anti-EGFR treatments in colorectal cancer. In particular, the KRAS G12C mutation, which accounts for approximately 3–4% of colorectal cancers (CRCs) and 12–14% of non-small cell lung cancers (NSCLCs), involves a cysteine substitution at codon 12. This has provided the opportunity to develop selective covalent inhibitors that trap the mutant protein in its inactive state. The first targeted therapies for KRAS G12C-mutant cancers comprise sotorasib and adagrasib, both of which have been authorized for use in patients with previously treated NSCLC and CRC. Nevertheless, despite the evidence of clinical activity for this class of agents, primary and acquired resistance, dose optimization, and toxicity management remain significant open challenges. In this review, we summarize recent advances in KRASG12C tumor biology and pharmacological targeting. We also provide additional insights to guide future efforts to overcome the limitations of the current approaches and implement the treatment of KRASG12C-mutant cancers.

## Simple Summary

KRAS G12C mutations represent a niche but important target in oncology, with implications across several tumor types. This review provides a comprehensive overview of current knowledge, spanning biological mechanisms, translational research, and clinical advances related to KRAS G12C-mutant cancers. By collecting and organizing all known evidence in a pancancer perspective, we aim to offer an integrated view of the progress made so far. Furthermore, we highlight the major challenges that remain to be addressed, such as resistance to therapies and optimization of treatment strategies, with the goal of guiding future research and improving patient outcomes.

## 1. Introduction

The KRAS oncogene is one of the most frequently mutated genes in human cancer, with mutations detected in approximately 30% of all malignancies [1]. Among the various KRAS mutations, the G12C variant stands out due to the unique biochemical properties provided by the cysteine residue which have enabled the development of selective covalent inhibitors [1,2,3].

The epidemiological distribution of KRAS G12C mutations varies significantly across different tumor types, reflecting the complex interplay between genetic susceptibility, environmental exposures, and tissue-specific oncogenic pathways [4]. In non-small cell lung cancer (NSCLC), KRAS G12C represents the most prevalent KRAS mutation subtype, accounting for approximately 11–16% of all NSCLC cases and 13% of lung adenocarcinomas specifically [5]. Among patients with KRAS-mutated NSCLC, the G12C variant comprises 29.4% to 41.5% of cases, making it the dominant KRAS mutation in this tumor type [6,7]. This mutation demonstrates a strong association with smoking history, occurring more frequently in current or former smokers (85% versus 56% in non-smokers) and showing a predominance in female and in older patients compared to other KRAS mutations [8,9].

In contrast, KRAS G12C mutations are detected in only 3–4% of all colorectal cancer (CRC) cases, representing approximately 6.4% of KRAS-mutated colorectal tumors [10,11]. This lower prevalence stands in stark contrast to other KRAS variants in CRC, where G12V (27.6%) and G12D (23.5%) mutations predominate [10,11]. Moreover, G12C mutation is more frequent in left-sided tumors and presents a consistent geographic distribution [4,12]. Pancreatic ductal adenocarcinoma (PDAC) presents yet another distinct pattern, with KRAS G12C mutations detected in only 2–3% of cases [13], despite the high (>90%) overall frequency of KRAS mutations in this tumor type [14]. In addition to these tumors, KRAS G12C mutations are also found in smaller proportions of other solid tumors, such as endometrial cancer and biliary tract cancers [15].

The rapid evolution in the management of KRAS G12C-mutated cancers necessitates the integration of both biological insights and clinical findings to establish a framework for future therapeutic developments. The transition from considering KRAS as “undruggable” to achieving regulatory approvals for multiple G12C-targeted agents within a span of less than a decade exemplifies the potential for translational research to transform patient care [16,17,18]. However, this progress has also revealed significant challenges, including primary and acquired resistance mechanisms, differential efficacy across tumor types, and the complexity of optimizing combination strategies. Understanding these biological foundations alongside clinical outcomes data is essential for developing next-generation approaches that can overcome current limitations and expand therapeutic benefits to broader patient populations [19].

This review examines the current state of KRAS G12C-targeted therapy from multiple perspectives. We begin with an analysis of the biological foundations underlying KRAS G12C function and the pharmacological principles enabling selective inhibition. Subsequently, we explore the clinical development pathway of current therapeutic agents, including sotorasib, adagrasib, and divarasib, with detailed examination of efficacy and safety data across different tumor types. We then address the critical challenge of resistance mechanisms, both primary and acquired, that limit the durability of current therapeutic approaches. Finally, we discuss emerging strategies on the horizon, including better patient selection and novel drug combinations, that hold promise for overcoming existing limitations and advancing the field toward more effective and durable therapeutic solutions for patients with KRAS G12C-mutated cancers.

## 2. Biology and Pharmacology of KRAS G12C

### 2.1. Biological Features of KRAS G12C-Mutant Cancers

KRAS constitutes a key cellular “switch”, able to integrate upstream stimuli coming from growth factor receptors and transmit the signals into multiple effector pathways to drive cellular growth and proliferation [20]. KRAS protein is able to self-limit the transmission of the signal through its intrinsic GTPase activity: while downstream effectors are engaged in its active form (GTP-bound), pathway activation is interrupted in the inactive GDP-bound form [21]. In contrast, due to the peculiar biochemical features induced by the aminoacidic changes, KRAS mutants present altered kinetics with regards to the exchange in GDP with GTP and/or the hydrolysis of GTP to GDP, eventually inflating their downstream signaling [22,23,24]. Interestingly, signaling pathways differ among different KRAS variants, with G12X mutations being more responsive to upstream receptor tyrosine kinase (RTK) activation and more conductive towards both MAPK and PI3K pathways compared to Q61X mutations [25].

In particular, the KRAS G12C mutant drives persistent activation of downstream signaling via GTP-bound RAS predominantly through the MAPK (RAF-MEK-ERK) pathway, while PI3K-AKT-mTOR pathway activation is less pronounced compared to other variants [26,27]. Importantly, G12C retains responsiveness to upstream RTK-mediated activation and intrinsic GTPase activity, cycling between active (GTP-bound) and inactive (GDP-bound) states. This unique property enables selective targeting of the GDP-bound conformation [28].

In addition to their biochemical properties, KRAS G12C cancers present a distinct co-mutation pattern that may influence the response to selective inhibitors and other treatments [29]. In particular, KRAS G12C-mutated NSCLC cases exhibit significantly higher rates of *STK11* (20.59% versus 5.95%) and *KEAP1* (15.38% versus 4.61%) alterations compared to other KRAS variants [8]. The presence of these concurrent mutations, particularly *KEAP1*, has been associated with reduced response rates to KRAS G12C inhibitors in NSCLC [8], while *STK11* mutations are associated with decreased immune sensitivity [30]. In CRC, in contrast, *STK11* mutations are not enriched in KRAS G12C-mutant tumors, while the rate of *PIK3CA* co-mutations is slightly increased compared to other *KRAS* mutants [10,31].

Moreover, KRAS G12C-mutant tumors are characterized by distinct immunological features. More broadly, it is known that KRAS hyperactivation can influence the immune tumor microenvironment through various mechanisms, including direct secretion of immune suppressive cytokines by tumor cells [32], recruitment and activation of T regulatory cells [33], PD-L1 hyperexpression [34], sensitization of cytotoxic T cells to activation-induced cell death [35], and suppression of interferon signaling [36,37]. For this reason, it is no wonder that selective KRAS G12C inhibition has been shown to significantly increase immune surveillance in preclinical models [38,39]. Additionally, in NSCLC the presence of G12C is associated with a higher average tumor mutational burden compared to non-G12C mutants or KRAS wild-type tumors, potentially impacting on immunotherapy sensitivity [40]. Importantly, the presence of *STK11* mutations decreases both the likelihood of PD-L1 positivity and the expected benefit from immune checkpoint inhibitors in KRAS G12C-mutant NSCLC [41]. Of note, KRAS G12C mutation does not cluster with MMRd/MSI status in CRC.

Finally, preclinical evidence suggests KRAS G12C-mutant tumors present peculiar metabolic features, such as decreased glucose and increased glutamine dependency, decreased lipogenesis, and increased autophagy, which may collectively play a role in dictating therapeutic response [42,43,44,45]. However, the clinical impact of this metabolic rewiring is difficult to ascertain given the lack of specific metabolic biomarkers.

### 2.2. Pharmacology of KRAS G12C Inhibitors

For decades, KRAS was deemed “undruggable” due to its smooth surface, picomolar GTP affinity, and lack of deep binding pockets [46]. The 2013 discovery of a previously unrecognized pocket beneath the effector binding switch-II region of the GDP-bound KRAS G12C protein revolutionized the field, enabling covalent targeting of the cysteine residue with a thiol-reactive warhead [16]. The consequential irreversible drug binding to the neomorphic cysteine residue leads to the protein being locked into the inactive GDP-bound state. As such, the first KRAS G12C inhibitors to reach clinical development are classified as RAS-OFF inhibitors, since they selectively stabilize inactive GDP-bound KRAS and thus rely on the G12C mutant’s retained GTPase activity. Conversely, RAS-ON inhibitors, currently in preclinical or early clinical development, bind to the active GTP-bound state and aim to overcome adaptive RTK-mediated reactivation [2]. Importantly, even though targeting of the active (GTP-bound) form of KRAS would be preferable, the accelerated GTPase kinetics of the G12C mutant compared to other variants explains why in presence of RAS-OFF inhibitors the G12C-mutant protein rapidly ends up being entirely trapped in the inactive state [2]. For the sake of conciseness, we will focus on the three selective inhibitors that are either approved or in advanced clinical testing: sotorasib, adagrasib, and divarasib.

Sotorasib is the first KRAS G12C inhibitor in its class to be FDA-approved, providing robust real-world safety and meaningful anticancer activity [47]. On the other hand, adagrasib has been optimized to achieve sustained pathway suppression with twice-daily dosing and CNS activity [48,49]. Divarasib stands out as the inhibitor with higher potency and greater selectivity for the G12C variant over wild-type KRAS compared to other inhibitors [50,51].

The main pharmacological features of sotorasib, adagrasib, and divarasib are summarized in Table 1.

## 3. Clinical Development of KRAS G12C Inhibitors

### 3.1. KRAS G12C Inhibitors in NSCLC

To date, three main molecules have been developed as KRAS G12C inhibitors: sotorasib, adagrasib, and divarasib. However, numerous other selective inhibitors are progressing towards clinical development [52]. In the following paragraphs, we will describe the clinical indications of these KRAS G12C inhibitors, their practical use in current clinical settings, and the regulatory approvals granted by agencies based on pivotal clinical trial data (Table 2).

Sotorasib is indicated as monotherapy for the treatment of adults with advanced NSCLC harboring a KRAS G12C mutation who have progressed after at least one prior line of systemic therapy. The U.S. Food and Drug Administration (FDA) granted accelerated approval to sotorasib in May 2021 based on the results of the CodeBreaK 100 trial, and the European Medicines Agency (EMA) followed with approval in 2022. Approval was primarily based on the results of the CodeBreaK 100 study, a multicenter, single-arm, open-label clinical trial that enrolled patients with locally advanced or metastatic KRAS G12C-mutated NSCLC who had progressed on or after prior systemic treatments [47]. In this trial, 124 patients received sotorasib at a dose of 960 mg orally once daily until disease progression or the emergence of unacceptable toxicity. The main efficacy outcomes were the objective response rate (ORR) and duration of response (DoR), as assessed by blinded independent central review (BICR) according to RECIST v1.1 criteria. The ORR was 36%, with a median duration of response of 10 months. The most common adverse reactions (≥20%) associated with sotorasib included diarrhea, musculoskeletal pain, nausea, fatigue, hepatotoxicity, and cough. The most frequently observed laboratory abnormalities (≥25%) were decreased lymphocytes, decreased hemoglobin, elevated aspartate aminotransferase (AST) and alanine aminotransferase (ALT), decreased calcium, increased alkaline phosphatase, increased urine protein, and decreased sodium levels. The recommended dose is 960 mg orally once daily, with or without food. This dosage was established based on clinical efficacy data as well as pharmacokinetic and pharmacodynamic modeling. As part of the accelerated approval, FDA required a post-marketing trial to assess whether a lower daily dose could achieve similar clinical efficacy. This investigation concluded that the 960 mg daily dose remains appropriate, fulfilling the original post-marketing requirement, although many oncologists consider this dose higher than necessary for safe and effective treatment. As a matter of fact, early-phase clinical trials testing doses ranges from 180 mg to 960 mg daily showed that tumor shrinkage and blood drug concentrations were similar across all dose levels. Higher doses did not yield greater efficacy but did intensify side effects, mostly gastrointestinal (diarrhea, nausea, vomiting, and mouth sores). In real-world data series, the ORR at the lowest doses were comparable to those at 960 mg, with overlapping confidence intervals for response and exposure. Moreover, a recent randomized phase 2 trial (NCT03600883) directly compared 240 mg vs. 960 mg. It showed only a modest numerical benefit for the higher dose, with an ORR of 32.7% vs. 24.8%, median PFS of 5.4 vs. 5.6 months, and OS of 13.0 vs. 11.7 months, while higher toxicity was observed at 960 mg. Other retrospective and real-world data found that dose reductions to 240–480 mg due to intolerance were common and did not appear to compromise efficacy; some analyses even suggested improved survival after dose reduction, though these findings are limited by small numbers and selection bias. However, in 2023, the FDA issued a Complete Response Letter following its review of the company’s supplemental New Drug Application for full approval, based on the CodeBreaK 200 trial [53]. The FDA concluded that an additional confirmatory trial would be necessary to support full approval, with a deadline for completion set for February 2028 [54]. This decision has sparked discussion within the medical community regarding the principles of accelerated approval. A viewpoint published in *JAMA Oncology* noted that while accelerated approval is intended for treatments addressing serious or life-threatening conditions based on surrogate endpoints, confirmatory trials should rigorously mirror the patient populations from the original approval [55]. In the case of sotorasib, discrepancies between trial design and real-world clinical settings were highlighted, particularly concerning dose optimization and indication specificity. Despite these regulatory hurdles and the limitations of the benefit demonstrated in clinical trials, sotorasib remains a significant advancement in oncology, and large clinical trials are still accruing patients for several indications.

Adagrasib underwent clinical development to treat adult patients with advanced NSCLC who have experienced disease progression after at least one prior systemic therapy. It was approved by the FDA in December 2022 and by the EMA in January 2024 as monotherapy for this indication. Adagrasib has been designed with several favorable properties, including a long half-life of approximately 23 h, dose-dependent pharmacokinetics, and the ability to penetrate the blood–brain barrier, an important feature given the high incidence of brain metastases in NSCLC. In early-phase studies, particularly the KRYSTAL-1 phase 1/2 trial, adagrasib demonstrated deep and durable responses, with promising progression-free survival (PFS) and overall survival (OS) in previously treated patients with KRAS G12C-mutated NSCLC, also including patients with untreated brain metastases [56,57]. The phase 3 KRYSTAL-12 study provided the key evidence supporting adagrasib’s efficacy and safety [58]. This randomized, open-label trial compared adagrasib to docetaxel in patients with locally advanced or metastatic KRAS G12C-mutated NSCLC who had received prior platinum-based chemotherapy and anti-PD-(L)1 therapy, either sequentially or concomitantly. Importantly, there was no required washout period between prior anti-PD-(L)1 therapy and initiation of study treatment. Patients randomized to docetaxel were allowed to cross over to adagrasib upon disease progression. In total, 301 patients were assigned to the adagrasib arm and 152 to the docetaxel arm. As of the data cutoff on 31 December 2023, with a median follow-up of 9.4 months, adagrasib demonstrated an improvement in PFS compared to docetaxel. The median PFS was 5.5 months for adagrasib versus 3.8 months for docetaxel. Similarly, the ORR according to BICR was significantly higher with adagrasib (31.9%) compared to docetaxel (9.2%). The median DOR was 8.3 months for adagrasib and 5.4 months for docetaxel. Treatment-related adverse events (TRAEs) were reported in 94.0% of patients treated with adagrasib and 86.4% of those treated with docetaxel. Grade ≥ 3 TRAEs occurred in 47.0% and 45.7% of patients, respectively. Treatment discontinuations due to TRAEs were less frequent with adagrasib (7.7%) compared to docetaxel (14.3%). The safety profile of adagrasib observed in KRYSTAL-12 was consistent with previous studies, with no new safety signals identified. These findings underline the clinical value of adagrasib and position it as a meaningful alternative to chemotherapy in this challenging patient population.

A promising new player in the KRAS G12C inhibitor class is divarasib, a covalent inhibitor with superior potency and selectivity. In August 2023, results from a phase 1 trial evaluating divarasib were published in the *New England Journal of Medicine* [50]. This trial included 137 patients with advanced or metastatic solid tumors (60 with NSCLC, 55 with CRC, and 22 with other solid tumors), all harboring the KRAS G12C mutation. Divarasib was administered orally once a day at doses ranging from 50 to 400 mg. The results were encouraging: no dose-limiting toxicities or treatment-related deaths were observed. Treatment-related adverse events occurred in 93% of patients, with 11% experiencing grade 3 events and 1% grade 4 events. Dose reductions due to toxicity were required in 14% of patients, while 3% discontinued the treatment permanently due to side effects. In the NSCLC cohort, a confirmed objective response was observed in 53.4% of patients, with a median PFS of 13.1 month. In the CRC cohort, the confirmed response rate was 29.1%, with a median PFS of 5.6 months. Notably, responses were also observed in patients with other solid tumors. Serial analysis of circulating tumor DNA revealed a reduction in the frequency of KRAS G12C mutations in patients with partial responses, and genomic alterations associated with resistance to divarasib were also identified. These results suggest that divarasib may offer more durable clinical responses and a longer PFS compared to current KRAS G12C inhibitors, such as sotorasib and adagrasib, in monotherapy. Given its promising antitumor activity and manageable safety profile, divarasib is considered a highly promising candidate for clinical use, both as a monotherapy and in combination with other antitumor therapies. Divarasib’s entry into the landscape of KRAS G12C inhibitors marks an interesting advancement in cancer therapy, particularly for tumors that are resistant to conventional treatments. However, data regarding CNS activity in clinical trials are still missing. Overall, due to its favorable pharmacodynamic profile and initial signs of efficacy, divarasib is generating significant interest in the scientific community, and there is hope that it will deliver more substantial benefits for patients with KRAS G12C-mutant cancers.

Recently, a meta-analysis explored potential biomarkers that could predict clinical outcomes in KRAS G12C inhibitor-treated NSCLC patients. Notably, the presence of a concurrent *KEAP1* mutation was associated with a lower ORR, suggesting that *KEAP1* status may influence the response to KRAS G12C inhibitors treatment. However, in this meta-analysis no significant differences in ORR were observed for mutations in *TP53* or *STK11* [59].

### 3.2. KRAS G12C Inhibitors in CRC

Since the first clinical reports, it was clear that the activity of KRAS G12C inhibitors in monotherapy was lower in CRC compared to NSCLC. As suggested, one peculiarity of the G12C variant consists in its responsiveness to upstream signaling by RTK.

This knowledge sparked hypotheses regarding the rapid feedback reactivation of the EGFR-MAPK pathway in response to KRAS inhibition in CRC, similarly to what was already reported for BRAF V600E inhibitors [60,61]. Amodio and colleagues showed that EGFR feedback activation is indeed the main reason explaining MAPK reactivation after KRAS blockade, prompting the initiation of clinical trials that included the combination of KRAS G12C inhibitors with anti-EGFR monoclonal antibodies [62], which have shown promising results.

The CodeBreaK 101 study evaluated sotorasib combined with panitumumab, an EGFR inhibitor, in patients with KRAS G12C-mutated CRC [63]. Early-phase results showed that this combination led to improved response rates and PFS compared to monotherapy with KRAS G12C inhibitors. This combination approach has led to the approval of sotorasib in combination with panitumumab by the FDA for the treatment of adult patients with KRAS G12C-mutated metastatic CRC who have previously undergone chemotherapy. This approval was based on the pivotal phase 3 CodeBreaK 300 trial, which showed that the combination of sotorasib and panitumumab resulted in superior PFS compared to standard-of-care (SOC) therapies like trifluridine/tipiracil or regorafenib in a chemorefractory CRC population [64]. In this trial, patients receiving the combination had a median PFS of 5.6 months, significantly longer than the 2 months observed in the SOC arm. The ORR in the combination group was 26%, while the SOC group had a response rate of 0%. However, the final OS analysis did not show a statistically significant difference between the arms, though the combination still demonstrated a clinically meaningful benefit in terms of PFS and ORR. The safety profile of the combination was manageable, with common adverse reactions including rash, dry skin, diarrhea, stomatitis, fatigue, and musculoskeletal pain. The most common grade 3–4 laboratory abnormalities included electrolyte imbalances, such as decreased magnesium, potassium, and calcium. Based on these results, the recommended dose for sotorasib is 960 mg orally once daily, while panitumumab is administered intravenously every 14 days.

In addition to sotorasib, adagrasib has also been investigated in combination with EGFR inhibitors. The KRYSTAL-1 study, which is investigating adagrasib combined with cetuximab, has yielded promising results in patients with advanced KRAS G12C-mutated CRC [65]. In a cohort of 94 patients, the combination showed a response rate of 34%, and a disease control rate of 85.1%. The median PFS was 7 months, and the median OS was 16 months. Notably, about 40% of patients who progressed after the combination therapy continued to receive additional treatment, which highlights the potential for long-lasting disease control even after the failure of the KRAS G12C inhibitor. Additionally, ctDNA analysis from 83 patients showed that liquid biopsy detected the KRAS G12C mutation in 80% of cases, and the clearance of the KRAS G12C mutation after the fourth cycle was predictive of response and prolonged survival. The KRYSTAL-1 study also underscored the importance of molecular selection in these combination therapies. The response rates were significantly higher in patients with KRAS G12C mutations detected by ctDNA analysis compared to those without. Although the study did not include a randomized control group, the promising results indicate that combining adagrasib with cetuximab can offer an effective treatment option for patients with KRAS G12C-mutant cancers. Ongoing phase 3 trial will further validate these findings and provide more robust data on the efficacy and safety of this combination [66]. Additionally, initial data are also available for the combination of divarasib and cetuximab [51]. In a phase 1b trial in CRC patients, the ORR was 62.5% (95% confidence interval: 40.6%, 81.2%) in KRAS G12C inhibitor-naive patients (*n* = 24), with a median duration of response of 6.9 months and an mPFS of 8.1 months (95% confidence interval: 5.5, 12.3). Explorative analyses on ctDNA showed that the decline in KRAS G12C variant allele frequency was associated with response and identified acquired genomic alterations at disease progression that may be associated with resistance [51].

An interesting review by Sayed et al. analyzed the efficacy and safety of KRAS G12C inhibitors in CRC. When used alone, sotorasib showed an ORR of 7.1 to 9.7%, with DCR ranging from 73.8% to 82.3% and a median PFS of 4 to 5.6 months. Combining sotorasib with panitumumab increased the ORR to 26.4%. Adagrasib combined with cetuximab showed a notable 42% ORR and a median PFS of 6.9 months, a significant improvement over the 19% ORR with adagrasib alone. Divarasib, when used alone, had a 36% ORR and an 85.5% DCR, with a median PFS of 5.6 months. The combination of divarasib with cetuximab raised the ORR to 62.5%, with a median PFS of 8.1 months. To sum up, the impact of KRAS G12C inhibitors in CRC differs based on the treatment approach. While single-agent therapies like sotorasib and adagrasib can help to stabilize disease progression, their relatively low ORRs suggest there is significant potential for improvement. Combining these inhibitors with EGFR inhibitors, such as cetuximab or panitumumab, offers better results in terms of response rates and PFS.

Overall, the combination of KRAS G12C inhibitors with EGFR inhibitors offers a highly promising therapeutic strategy for KRAS G12C-mutant CRC. These combinations overcome resistance mechanisms, enhance the efficacy of treatment, and improve patient outcomes [67]. The results from ongoing trials will likely support the potential of these combinations to become an integral part of the treatment of KRAS G12C-mutant malignancies.

### 3.3. Overall Efficacy Data in Solid Tumors

Two recent meta-analyses investigated the activity of KRAS G12C inhibitors across cancers. The first one analyzed 18 studies including all solid tumors [59]. The pooled data demonstrated an overall ORR of 31% and a DCR of 86%. This suggests that a significant proportion of patients experience clinical benefit from KRAS G12C inhibitor treatment. Subgroup analysis revealed that NSCLC patients had a higher ORR (35%) compared to CRC (24%) and other solid tumor patients (24%), although the DCR remained similar across tumor types. For NSCLC, the DCR was 83%, for CRC it was 88%, and for other solid tumors, it was 91%. The analysis also highlighted the differences in efficacy between monotherapy and combination treatments. KRAS G12C inhibitor monotherapy showed an ORR of 29% and a DCR of 85%, while combination therapies yielded a slightly better ORR of 34% and a DCR of 87%. This indicates that combining KRAS G12C inhibitors with other therapeutic strategies may improve the clinical outcomes of treatment. Combination therapies were further categorized based on the targeting strategy, including upstream KRAS molecules (such as EGFR and SHP2), downstream KRAS molecules (like MEK), and ICIs targeting PD-1/PD-L1. Among these combinations, co-targeting upstream KRAS molecules (EGFR, SHP2) led to the highest ORR of 37% and a DCR of 89%. Combinations with downstream KRAS molecules had a lower ORR (14%) and DCR (83%), while ICIs produced an ORR of 39% and a DCR of 86%. In the second meta-analysis, Dang et al. showed that KRAS G12C inhibitors presented an encouraging clinical activity in advanced solid tumors, particularly in heavily pretreated patients. Monotherapy with these agents achieved a good ORR, DCR, and PFS, and early responses were relatively durable. In NSCLC, outcomes were better compared to CRC and other tumors, although OS benefits remain uncertain. KRAS G12C inhibitors combined with EGFR inhibitors have shown improved efficacy over standard care in CRC [68,69].

Beyond NSCLC and CRC, KRAS G12C inhibitors were also tested in other solid tumors, particularly in PDAC and biliary tract cancers (BTCs). In PDAC, sotorasib showed partial responses in roughly 21% of heavily pretreated patients, with a disease control rate of 84%, median PFS of 4 months, and median OS of 6.9 months. Adagrasib produced higher response rates (33–50%) and a median PFS of 5.4–6.6 months in the KRYSTAL-1 trial, with similar tolerability. Adagrasib was also tested in BTCs, showing an ORR of 41.7%, median PFS of 8.6 months, and OS of 15.1 months. For both agents, efficacy was clearly below what has been achieved in NSCLC, despite being meaningfully greater than that of standard second-line chemotherapy.

The relative lower activity in pancreatic and biliary tract cancers is generally associated with inherent tumor heterogeneity and significant co-mutations in resistance genes or with strong pathway redundancy. Taken together, these results suggest that tissue-specific features should not be overlooked, and that a tissue-agnostic approach to treating KRAS G12C-mutant cancers should be avoided.

### 3.4. Toxicity Data and Quality of Life

The toxicity profile of sotorasib and adagrasib has been characterized in multiple cohorts, with several meta-analyses published on the topic [59]. Globally, the overall incidence of any-grade TRAEs was 80–95% across the total population, while the pooled incidence of grade ≥ 3 adverse events was 24–29%, with a higher rate reported for adagrasib (40%). The most common grade ≥ 3 AEs across studies included increased ALT, increased AST, and prolonged QT interval on ECG. Toxicity was lower when co-targeting with KRAS upstream molecules (28%) than that for combination with ICIs (50%). The most frequently reported any-grade adverse events (≥10%) included diarrhea, nausea, vomiting, fatigue, ECG QT prolongation, elevations in AST and ALT, increased amylase and blood creatinine (or acute kidney injury), decreased appetite, and anemia. Less common (1–10%) events included elevated alkaline phosphatase, increased lipase, dysgeusia, peripheral edema, hyponatremia, abdominal pain, pneumonitis, decreased lymphocyte count, and decreased neutrophil count. Among grade 3 or higher adverse events, the most common were significant elevations of ALT and AST, diarrhea, ECG QT prolongation, increased lipase, anemia, fatigue, elevated alkaline phosphatase, increased amylase, and hyponatremia. Less frequent severe events (0.1–1%) included acute kidney injury, pneumonitis, lymphopenia, appetite loss, nausea, neutropenia, abdominal pain, and vomiting. Subgroup analyses showed that most any-grade adverse events were more frequent with adagrasib compared to sotorasib. However, for grade 3 or higher events, the pattern was more mixed, with some side effects being more common with adagrasib and others not showing a clear difference [68]. Importantly, a recent report suggests that the relationship between sotorasib doses and toxicity should be closely monitored, in order to optimize its use [70].

While not included in meta-analyses, divarasib’s toxicity profile is similar to that of sotorasib and adagrasib. In particular, in the recent phase 1 trial, 93% of patients experienced TRAEs, mostly grades 1–2. Grade 3 events occurred in 11%, and grade 4 in 1%, with no grade 5 events. The most common AEs were nausea (up to 78%), vomiting (63%), diarrhea (60%), and fatigue (27%). Dose reductions occurred in 14% and treatment discontinuation due to toxicity in 3%. Regarding the toxicity profile of newer, more potent, selective KRAS G12C inhibitors (discussed in Paragraph 5.1), it appears to be similar to that reported for first-in-class molecules. The most common TRAEs involve the gastrointestinal (GI) tract, with nausea, vomiting, diarrhea, and liver toxicity of all grades being reported in more than 50% of cases.

Patient-reported outcomes (PROs) and health-related quality of life (HRQoL) data for KRAS G12C inhibitors are primarily available for sotorasib, particularly in NSCLC. In the CodeBreaK 100 trial, disease-related symptoms and HRQoL were evaluated as exploratory endpoints with the European Organization for Research and Treatment of Cancer Quality-of-life Questionnaire Core 30 (EORTC QLQ-C30) and its lung cancer module, EORTC Quality-of-life Questionnaire Lung Cancer 13 (QLQ-LC13). Sotorasib demonstrated maintenance or improvement in global health status, physical functioning, and key lung cancer symptoms such as cough, dyspnea, and chest pain. Most patients reported minimal issues from treatment-related side effects [71]. In the phase 3 CodeBreaK 200 trial, PROs were considered as secondary and exploratory endpoint. The PRO measures used were EORTC QLQ-C30, EORTC QLQ-LC13, question GP5 from the Functional Assessment of Cancer Therapy Tool General Form (FACT-G GP5), PRO-Common Terminology Criteria for Adverse Events (PRO-CTCAE), and 5-level EuroQOL-5 dimensions (EQ-5D-5L) including a visual analog scale (EQ-5D VAS). Patients receiving sotorasib reported less severe symptoms and maintained quality of life, whereas those on docetaxel experienced a decline. Specifically, sotorasib-treated patients were less bothered by side effects and had fewer symptoms interfering with daily activities [72]. Currently, there is limited data on PROs and HRQoL for other KRAS G12C inhibitors like adagrasib. Further studies are needed to evaluate the impact of these treatments on patient HRQoL.

## 4. Resistance to KRAS G12C Inhibitors

**Figure 1:**
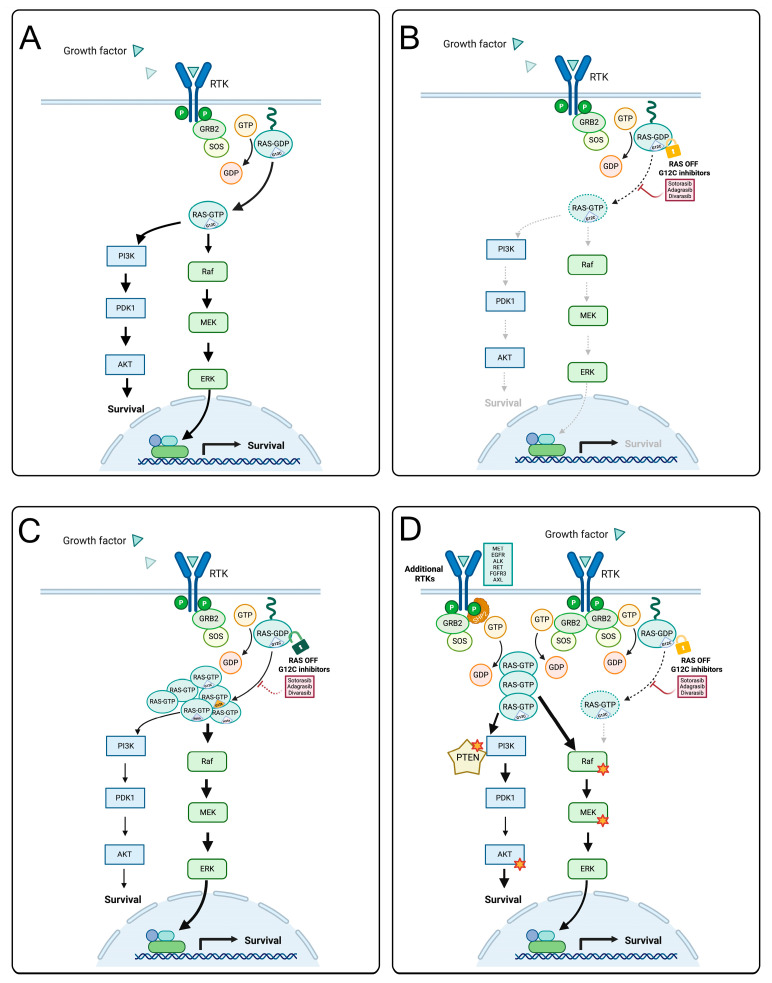
KRAS signaling pathway, adaptation and resistance mechanisms to G12C-specific inhibitors. (**A**). KRAS G12C-mutant cancers display an increased downstream activation of the survival pathways through the RAS-MAPK and the RAS-PI3K axes. (**B**) In the presence of selective G12C inhibitors (sotorasib, adagrasib, and divarasib), the KRAS G12C protein is locked in the GDP-bound inactive state, leading to a dramatic reduction in downstream signaling. (**C**) On-target resistance mechanisms, consisting of KRAS amplification and/or the emergence of additional KRAS variants that are insensitive to the activity of the G12C-specific inhibitor, restore the downstream signaling pathways. (**D**) Off-target resistance mechanisms consist of the activation of bypass signaling pathways upstream or downstream of the KRAS G12C protein through acquired gene amplification or mutation (sparks). See the text for details. Figure generated with BioRender™.

Despite the sustained pharmacological activity of selective KRAS G12C inhibitors in suppressing downstream MAPK signal transduction (Figure 1A,B), the problem of both primary and acquired resistance became evident in the early phases of clinical development, limiting the clinical activity of this new class of drugs.

Primary (intrinsic) resistance arises before or at the start of treatment and is driven by several distinct molecular features. In particular, co-occurring amplifications in genes such as *HER2* and *PIK3CA* or inactivation of *STK11*, *CDKN2A*, *TP53*, or *PTEN* can promote pathway bypass or activate parallel oncogenic circuits, diminishing the efficacy of KRAS G12C inhibition. Additionally, as already discussed, RTK/MAPK pathway reactivation is a particularly prominent mechanism in CRC, where EGFR-mediated reactivation is a key driver of primary resistance.

Regarding acquired resistance, mechanisms can be divided into on-target and off-target events (Figure 1C,D and Table 3). On-target events involve high-level genetic amplification of the *KRAS* gene and/or the emergence of novel mutations in the target protein that impair the inhibitor binding without compromising the signaling activity [73]. Examples of acquired KRAS mutations include secondary codon 12 KRAS mutations (G12D/R/V/W), codon 13 and codon 61 alterations (G13D, Q61X), and mutations at the switch-II pocket, corresponding to the drug binding sites (R68S, H95D/Q/R, and Y96C) (Figure 1C) [73]. The presence of non-G12C KRAS variants renders the inhibitor unable to block KRAS-dependent signaling, eventually resulting in treatment resistance.

Off-target resistance events, on the other hand, involve the activation of bypass signaling pathways up- or downstream of the KRAS G12C protein [74]. These events can be the result of signaling adaptation, as in the case of EGFR feedback reactivation in CRC [62], or the consequence of genetic events such as *MET* amplification [75]. Other events that have been associated with resistance include loss-of-function mutations in *NF1* (a negative regulator of RAS activity) and *PTEN* (a negative regulator of PI3K signaling pathway)*,* activating mutations in *NRAS*, *BRAF*, *MAP2K1*, *RAF1*, and *FGFR3*, as well as oncogenic fusions of *ALK*, *RET*, *BRAF*, *RAF1*, and *FGFR3* (Figure 1D) [73]. Interestingly, multiple alterations are found in up to one fifth of the cases [73,74,76]. The acquisition of resistance mutations that emerge as a consequence of the therapeutic pressure may be fostered by the adaptive mutability process, a known consequence of oncogenic deprivation that has been reported in multiple tumors treated with targeted therapy, including NSCLC and CRC [77,78]. In general, every mutational event able to activate RTKs or downstream effectors (such as BRAF, MEK, and MAPK) can mediate resistance to selective KRAS G12C inhibitors, eventually converging on RAS-MAPK pathway reactivation, as previously described for adaptive resistance to the inhibition of other nodes of the same pathway [61,79,80]. Interestingly, it has been previously reported that the protein tyrosine phosphatase SHP2 constitutes a vulnerability in KRAS-mutated tumors, as it is involved in mitogenic signaling from multiple RTKs to the RAS-MAPK pathway [81]. Unsurprisingly, vertical pathway inhibition using the concurrent inhibition of KRAS G12C and SHP2 has proven effective in overcoming adaptive feedback resistance across multiple tumor histologies in preclinical studies [82]. As a direct consequence of this preclinical observation, several trials are evaluating combinations of KRAS G12C inhibitors with SHP2 inhibitors [57,83,84].

In addition to SHP2, a full characterization of the essential mediators of the cellular adaptation to KRAS inhibition—the so-called *collateral dependencies*—has been performed [85]. These mediators include RTKs like EGFR and FGFR, as well as tumor cell survival pathways like AXL, PI3K, or CDK4/6 [85]. Of note, the same mediators of acute and long-term response to G12C inhibitors were uncovered by using a proteomic approach, underlining the redundancy of cellular adaptation mechanisms [86]. Since the loss of these mediators enhances cellular susceptibility to pharmacological KRAS G12C inhibition, this notion constitutes the biological basis for several combination strategies [85,86].

More recently, the type of feedback reactivation of survival pathways in cancer cells upon KRAS G12C inhibition has been correlated with the transcriptional features [87]. In one paper by Solanki and colleagues, NSCLCs with an epithelial gene signature tended to upregulate ERBB2/3 signaling in response to KRAS repression, while cancers with a mesenchymal signature relied more on FGFR1 and AXL signaling [87]. This observation suggests that different transcriptional programs may cause resistance to KRAS G12C inhibitors. Indeed, both epithelial–mesenchymal transition and adeno-squamous histological transformations have been demonstrated to play an active role in G12Ci resistance in NSCLC [88,89].

Moving to the resistance to combined EGFR + KRAS G12C blockade in CRC, even in this case most mechanisms converge on RAS-MAPK pathway reactivation either by de novo mutations or by parallel signaling through other RTKs [90]. Moreover, KRAS G12C amplification has also been reported for the combination of sotorasib and panitumumab [91]. Interestingly, among the acquired mutations detected in ctDNA samples from patients progressed to combination therapy, some belonged to cell cycle control, DNA methylation, and DNA damage response pathways, as in the case of *TP53*, *DNMT3A*, and *LRP1B* mutations, found in 34%, 17%, and 11% of patients at the moment of disease progression [91]. While correlative, these results suggest that other events different from RTK-MAPK reactivation are at play and may account for the failure of the vertical suppression approach. Of note, multiple mechanisms are found in most cases of resistance, highlighting the subclonal nature of the genetic events [90,91].

Altogether, understanding the trajectories leading to treatment resistance paves the way for combination strategies aimed at extending the benefits of KRAS G12C inhibition in cases of primary or acquired resistance [92].

## 5. Open Questions and Future Challenges

While KRAS G12C inhibitors offer new hope for patients with previously difficult-to-treat cancers, the rapid co-evolution of the biological and clinical scenario creates new challenges. The development of newer, more potent, inhibitors prompts the identification of more reliable biomarkers. Similarly, the heterogeneity of resistance patterns across tissue types calls for a more personalized approach to therapy, while the intersection between KRAS signaling and other biologically relevant pathways suggests novel combinations could provide additional benefit. Moreover, the clinical development of newer inhibitors and novel combination strategies will require careful evaluation of the balance between efficacy and tolerability.

### 5.1. Novel Inhibitors and Combinations

The enthusiasm deriving from the first clinical results of selective KRAS G12C inhibitors has prompted the development of a plethora of different covalent RAS-OFF inhibitors with enhanced pharmacodynamic properties [93], such as divarasib [94], garsorasib [95], MK1084 [96], or olomorasib [97], whose main features are summarized in Table 4.

While their clinical performance is emerging to be at least comparable to that of the first inhibitors to reach the market, it is also expected that adaptation and resistance mechanisms will be similar [93]. Indeed, it must be noted that while compounds with enhanced pharmacodynamic or pharmacokinetic features are needed and several clinical trials are ongoing, they mostly represent incremental progress. Conversely, recently developed RAS-ON inhibitors have proven to be active in tumors progressed upon treatment with selective G12C RAS-OFF inhibitors, as they suppress RAS reactivation sustained by feedback mechanisms or KRAS state adaptation [103,104]. In a phase 1 study, daraxonrasib (RMC-6236), an oral, multi-selective RAS-ON inhibitor, demonstrated efficacy and manageable safety in patients with PDAC harboring KRAS G12X or other RAS mutations (NCT05379985). In particular, RMC-6236 presented an ORR of 25–29% in pretreated patients with KRAS mutant PDAC, while the most common TRAEs were skin/mucosal toxicity (rash, paronychia, mucosal inflammation, peripheral edema, and stomatitis) and gastrointestinal events (diarrhea, nausea, vomiting, and decreased appetite). However, despite these results holding promise for achieving unprecedented progress in patients with any RAS mutation, clinical trials exploring RAS-ON inhibitors are still underway and results are still immature at the present stage.

On the other hand, combination strategies of KRAS G12C inhibitors with standard-of-care or investigational drugs represent a viable option that is being investigated extensively in clinics. The goal of these combinations is not only to increase response rates but also to prevent or delay the onset of resistance, thereby achieving more durable disease control. While the scientific rationale for combination therapies is compelling, their clinical development faces important challenges. Toxicity represents the most clinically relevant issue, since combining multiple agents often increases the risk of adverse events. For example, adding EGFR inhibitors can exacerbate dermatologic toxicities, while SHP2 inhibitors may cause hematologic side effects. Importantly, dose optimization could help in minimizing toxicities, though the balance with treatment efficacy is sometimes complex. Another challenge consists in patient selection: biomarkers are indeed urgently needed to identify the patients most likely to benefit from specific combinations and to monitor the emergence of resistance. Another non-trivial issue of the combinations is their cost and financial toxicity, which may limit their accessibility and use in routine clinical practice.

Chemotherapy combinations have been explored in both NSCLC and CRC. In NSCLC, a phase 1b/2 clinical trial investigating carboplatin/pemetrexed with sotorasib has shown encouraging results, with a high response rate in the first and second line, despite an increase in grade 3–4 hematologic malignancies leading to discontinuation [63]. However, the clinical relevance of chemotherapy combinations is probably higher in CRC, for which chemotherapy/anti-EGFR regimens are already part of standard care. Early results from the phase 1b trial CodeBreaK 101 support the development of this approach [105,106], with a manageable toxicity and a 75% ORR for the FOLFIRI/panitumumab/sotorasib combination having prompted the initiation of a large phase 3 trial testing this regimen in the first-line setting [107].

Additionally, given the dramatic interplay between KRAS-mutated cancer cells and the immune-suppressive microenvironment, the addition of KRAS G12C inhibitors to (chemo) immunotherapy treatment constitutes a biologically sound approach [52]. As explained above, by combining KRAS G12C inhibitors with anti-PD-1/PD-L1 therapies, it may be possible to stimulate a stronger immune response.

For example, the KRYSTAL-7 trial, combining adagrasib with pembrolizumab (an anti-PD-1 antibody) showed promising results in patients with advanced KRAS G12C-mutant NSCLC. While the combination is more effective in the case of tumors with PD-L1 > 50%, the combination led to higher response rates and longer PFS compared to monotherapy with adagrasib alone regardless of the PD-L1 status [108]. Beyond adagrasib, sotorasib, divarasib, and more recent G12C selective inhibitors are also currently being tested in combination with first-line immunotherapy, suggesting that this will likely become the preferential setting for the treatment of NSCLCs harboring a KRAS G12C mutation [109,110,111].

Moreover, other innovative rationally designed combinations are being progressively evaluated. In more detail, some research studies have focused on finding synthetic lethal approaches for KRAS G12C tumors [86]. The biological principle involves exploiting specific vulnerabilities induced by KRAS G12C mutation in cancer cells. One of the best characterized dependencies involves cell cycle regulation, potentially explaining why the combination of selective G12C inhibitors with CDK4/6 inhibitors presents a synergistic activity in preclinical models of pancreatic cancer and NSCLC [112,113]. This biological concept paves the way for clinical investigations with similar combinations, such as the KRYSTAL-12 study that is investigating adagrasib and palbociclib in KRAS G12C-mutant tumors [114].

Altogether, despite the continuous generation of more potent and selective inhibitors for KRAS G12C-mutant cancers, the current developmental landscape suggests that the progressive integration of G12C inhibitors into well-established treatment settings will represent the preferred path to maximize their therapeutic value.

### 5.2. Novel Biomarkers and Patient Selection for KRAS G12C-Mutant Tumors

Since the earliest days of the clinical development of selective G12C inhibitors, it has been clear that treatment response is heterogeneous, suggesting that not all KRAS G12C-mutant tumors are equally sensitive to a blockade of the KRAS pathway. As a matter of fact, the concept of KRAS-independency due to either collateral dependencies and/or different transcriptional phenotypes has been described in both CRC and NSCLC [115,116]. This points to the importance of properly selecting the right treatment or combination basing on the molecular features of each KRAS G12C-mutant cancer.

Genetic biomarkers, in particular, remain foundational. Their assessment is increasingly performed using next-generation sequencing (NGS) of tumor tissue or, more recently, of circulating tumor DNA (ctDNA).

Tissue genetic analysis is particularly useful to identify co-mutational patterns associated with decreased activity of the KRAS G12C selective inhibitors. In NSCLC, the predictive role of *KEAP1*, *STK11,* and *TP53* mutations has already been extensively assessed preclinically and retrospectively in clinical cohorts [8,117,118,119]. However, recent clinical evidence also confirms the strong impact of *STK11* and *TP53* co-mutations on sotorasib activity in a prospectively generated cohort from the Lung-MAP platform trial [120], suggesting these biomarkers are probably ready to be more widely incorporated into clinical practice. In colorectal and pancreatic cancers, additional co-mutations in *KRAS* (non-G12C alleles), *EGFR*, *PIK3CA*, *BRAF*, and *MAP2K1* are frequently detected at baseline and are linked to primary resistance, underscoring the importance of comprehensive NGS at diagnosis [121,122]. Currently, however, no genetic biomarker except for the presence of the G12C variant in KRAS can be considered validated for selecting the patients most likely to benefit from G12C selective inhibition. In this framework, it would be desirable to validate these genetic biomarkers in larger prospective patient cohorts and—ideally—in randomized trials.

On the other hand, circulating dynamic biomarkers, particularly ctDNA, are increasingly used for real-time monitoring, enabling the early assessment of treatment response and the detection of emerging resistance mutations. For instance, a rapid and deep decline in KRAS G12C ctDNA levels after initiation of targeted therapy correlates with radiographic response and longer progression-free survival, while incomplete clearance or rising ctDNA can signal early resistance or disease progression [123,124]. However, the most relevant application of ctDNA testing consists in the dynamic detection of genetic mechanisms of acquired resistance—such as secondary mutations in *KRAS*, *NRAS*, *BRAF*, *EGFR*, and *MAP2K1* or the loss of *CDKN2A/B* [91,121,125]. While ctDNA is mainly used for research purposes, its full integration in clinical practice could guide the selection of patients for combination strategies designed to be more effective, in addition to providing relevant biological information about tumor evolution and heterogeneity [126].

In addition to genetic factors, research settings increasingly recognize the role of other molecular characteristics in predicting treatment response. Notably, transcriptional biomarkers are emerging as key determinants of therapeutic outcomes. Recent large-scale transcriptomic analyses have defined three robust subtypes of KRAS G12C-mutant NSCLC (KC, KL, and KP) with distinct gene expression signatures, co-mutation patterns, immune microenvironments, and clinical outcomes. For example, low TTF-1 (NKX2.1) expression and high NRF2 activity (KC subtype) are associated with poor response to sotorasib, while KP tumors (enriched for *TP53* mutations and T-cell inflammation) show better outcomes. These transcriptional signatures can be assessed by RNA sequencing or immunohistochemistry and may guide therapeutic intensification or trial stratification [125].

Pathomics—the use of computational pathology and image analysis—has the potential to further refine patient stratification by linking histomorphologic features (such as mucinous differentiation or TTF-1 status) to molecular subtypes and clinical outcomes. For instance, reduced TTF-1 expression, readily assessable by routine immunohistochemistry, is now recognized as a putative negative prognostic biomarker in KRAS G12C-mutant NSCLC treated with sotorasib [29,125]. Moreover, artificial intelligence (AI) and machine-learning (ML) approaches are being applied across these biomarker domains. Integrative analyses combining genomics, transcriptomics, and pathomics using AI are poised to further personalize therapy and predict resistance in KRAS G12C-driven tumors. For instance, a machine-learning-based patient classifier has been recently developed to predict response to sotorasib in NSCLC based on the biological features identified in patient specimens [127].

In summary, the rapid co-development of new therapeutic strategies that are integrated with genetic, transcriptional, pathologic, and circulating biomarkers—augmented by AI and ML tools—will probably redefine the way we manage KRAS G12C-mutant cancers, enabling personalized selection, early response assessment, and adaptive therapeutic strategies.

## 6. Conclusions

The therapeutic quest to treat KRAS G12C-mutant cancers with selective inhibitors started less than ten years ago has already provided multiple advancements towards a progressive optimization based on clinical and biological breakthroughs. Future research on KRAS G12C inhibitors will likely focus on several key areas: (1) integrating molecular biomarkers into clinical decision-making; (2) optimizing the activity and the toxicity/QoL profile of combination treatments, with the aim of integrating G12C inhibitors earlier in disease management; (3) intercepting and selectively targeting resistance through rationally designed combinations to overcome adaptive mechanisms of resistance. Over the next ten years, we will probably see unprecedented advancements in multiple areas, spreading the “KRAS revolution” to other RAS variants and to an even greater number of clinical settings.

## Figures and Tables

**Table 1:** Relevant pharmacological parameters for sotorasib, adagrasib, and divarasib.

Parameter	Sotorasib	Adagrasib	Divarasib
**Target** **Residence**	Irreversible covalent binding	Irreversible covalent binding	Irreversible covalent binding
**Half-Life**	5.4 h	23 h	18–24 h
**CNS** **Penetration**	Limited	High (brain/plasma ratio 0.3–0.6)	N/A
**Potency** **(KRAS G12C IC_50_)**	21 nM	2.5 nM	0.6 nM

**Table 2:** Indications and approval status for the KRAS G12C inhibitors available in clinical practice (as of July 2025).

KRAS G12C Inhibitors	Indication	Approved by	Clinical Trial (Phase 3)
Sotorasib	Treatment of adults with advanced NSCLC harboring a KRAS G12C mutation who have progressed after at least one prior line of systemic therapy	FDA, EMA	CodeBreaK 100
Adagrasib	Treatment of adult patients with advanced NSCLC who have experienced disease progression after at least one prior systemic therapy	FDA, EMA	KRYSTAL-12
Sotorasib + Panitumumab	Treatment of adult patients with KRAS G12C-mutated metastatic CRC who have previously undergone chemotherapy	FDA	CodeBreaK 300

**Table 3:** Genetic resistance mechanisms to KRAS G12C inhibitors and their prevalence rates in NSCLC and CRC, based on large clinical and genomic studies. Prevalence rates can vary among studies; data reflect major pooled multicenter analyses.

Resistance Mechanism	NSCLC Prevalence (%)	CRC Prevalence (%)	Notes
Secondary KRAS mutations	20–25	30–40	Y96C/D/S, G13D, Q99, etc.
KRAS amplification	22	Not well reported	More frequent in NSCLC than CRC
RAS/MAPK pathway alterations	26	69	Includes *NRAS*, *BRAF*, *MAP2K1*, *EGFR*, *MET*, *HER2*, *PI3KCA*, etc.
Multiple concurrent events	23	Higher	Often a combination of the above mutations
EGFR pathway activation	10–15	Up to 30	Key adaptive resistance driver, especially in CRC
MET amplification	1–6	Rare	Notable in NSCLC, less frequent in CRC
PI3K pathway activation	8–10	15–20	Includes *PI3KCA*, *PTEN*, *mTOR* alterations

**Table 4:** Key clinical and developmental features of newer KRAS G12C inhibitors.

	Divarasib (GDC-6036)	Garsorasib (D-1553)	MK-1084	Olomorasib (LY3537982)
**Current clinical phase**	Phase 3 (NSCLC) Phase 1/2 (CRC)	Phase 3 (NSCLC) Phase 2 (CRC)	Phase 3 (NSCLC and CRC)	Phase 2/3 (NSCLC) Phase 1/2 (CRC)
**Key trial results**	NSCLC [50]: ORR: 53.4% mPFS 13.1 mo CRC [51]: ORR: 29% (monotherapy), 62.5% (+cetuximab) mPFS 5.6 mo (monotherapy), 8.1 mo (+cetuximab)	NSCLC [98]: ORR: 50% CRC [99]: ORR: 19.2% (monotherapy), 45.2% (+cetuximab) mPFS: 5.5 mo (monotherapy), 7.5 mo (+cetuximab)	NSCLC [100]: ORR: 38% mPFS: 8 mo CRC [101]: ORR: 36% (monotherapy), 50% (+cetuximab)	Non-CRC [97]: ORR: 39–40% mPFS: 4–9 mo CRC [102]: ORR: 38–44% (+cetuximab) mPFS: 6.6–7.5 mo
**Relevant features**	High potency, high selectivity, long half-life [50,94]	High selectivity, high CNS penetration [95] Breakthrough designation in China	Macrocyclic structure for improved selectivity and PK [96]	>90% target occupancy [97] Active in G12Ci-pretreated NSCLC [97]

## Data Availability

Not applicable.
